# Adenosine A2b receptor promotes progression of human oral cancer

**DOI:** 10.1186/s12885-015-1577-2

**Published:** 2015-07-31

**Authors:** Hiroki Kasama, Yosuke Sakamoto, Atsushi Kasamatsu, Atsushi Okamoto, Tomoyoshi Koyama, Yasuyuki Minakawa, Katsunori Ogawara, Hidetaka Yokoe, Masashi Shiiba, Hideki Tanzawa, Katsuhiro Uzawa

**Affiliations:** 1 Department of Oral Science, Graduate School of Medicine, Chiba University, 1-8-1 Inohana, Chuo-ku, Chiba 260-8670 Japan; 2 Department of Dentistry and Oral-Maxillofacial Surgery, Chiba University Hospital, 1-8-1 Inohana, Chuo-ku, Chiba 260-8670 Japan; 3 Department of Oral and Maxillofacial Surgery Research Institute, National Defense Medical College Hospital, 3-2, Namiki, Tokorozawa-shi, Saitama 359-8513 Japan; 4 Department of Medical Oncology, Graduate School of Medicine, Chiba University, 1-8-1 Inohana, Chuo-ku, Chiba 260-8670 Japan

## Abstract

**Background:**

Adenosine A2b receptor (ADORA2B) encodes an adenosine receptor that is a member of the G protein-coupled receptor superfamily. This integral membrane protein stimulates adenylate cyclase activity in the presence of adenosine. Little is known about the relevance of ADORA2B to human malignancy including oral squamous cell carcinoma (OSCC). We aimed to characterize the expression state and function of ADORA2B in OSCC.

**Methods:**

The ADORA2B expression levels in nine OSCC-derived cells were analyzed by quantitative reverse transcriptase-polymerase chain reaction and immunoblotting analyses. Using an ADORA2B knockdown model, we assessed cellular proliferation and expression of hypoxia-inducible factor1α (HIF-1α). We examined the adenosine receptor expression profile under both normoxic and hypoxic conditions in the OSCC-derived cells. In addition to *in vitro* data, the clinical correlation between the ADORA2B expression levels in primary OSCCs (*n* = 100 patients) and the clinicopathological status by immunohistochemistry (IHC) also was evaluated.

**Results:**

*ADORA2B* mRNA and protein were up-regulated significantly (*p* < 0.05) in seven OSCC-derived cells compared with human normal oral keratinocytes. Suppression of *ADORA2B* expression with shRNA significantly (*p* < 0.05) inhibited cellular proliferation compared with the control cells. HIF-1α also was down-regulated in ADORA2B knockdown OSCC cells. During hypoxia, *ADORA2B* expression was induced significantly (*p* < 0.05) in the mRNA and protein after 24 hours of incubation in OSCC-derived cells. IHC showed that ADORA2B expression in primary OSCCs was significantly (*p* < 0.05) greater than in the normal oral counterparts and that ADORA2B-positive OSCCs were correlated closely (*p* < 0.05) with tumoral size.

**Conclusion:**

Our results suggested that ADORA2B controls cellular proliferation via HIF-1α activation, indicating that ADORA2B may be a key regulator of tumoral progression in OSCCs.

**Electronic supplementary material:**

The online version of this article (doi:10.1186/s12885-015-1577-2) contains supplementary material, which is available to authorized users.

## Background

The adenosine receptors consists of four members that belong to the G protein-coupled receptor superfamily, including A1, A2A, A2B and A3 [1]. Activation of ADORA2A and ADORA2B causes activation of adenylate cyclase and phospholipase C [2]. ADORA2B is found in many different cellular types and requires higher concentrations of adenosine for activation than the ADORA1, 2A, and 3 subtypes [2]. ADORA2B mainly is located peripherally and is involved in inflammation and immune response [3]. ADORA2B is not stimulated by physiologic levels of adenosine and therefore may play an important role in pathophysiologic conditions associated with massive adenosine release. Such conditions occur in tumors when hypoxia is commonly observed [4, 5]. Moreover, ADORA2B was overexpressed in colorectal carcinomas grown under hypoxic conditions [6]. Adenosine was reported to modulate proliferation of cancer cells by a hypoxia-inducible factor1α (HIF-1α)-dependent mechanism [7]. HIF-1 expression and activity also are regulated by important signal transduction pathways including those involving extracellular regulated kinase 1/2 (ERK1/2) and Akt [8]. We hypothesized that ADORA2B may be associated with cancer progression.

Growing evidence indicates that the adenosine-receptor pathway also may be a promising therapeutic target in a wide range of conditions, including cerebral and cardiac ischemic diseases, sleep disorders, immune and inflammatory disorders, and cancer [9]. However, the expression status and function of ADORA2B in oral squamous carcinoma (OSCC) have not been characterized fully.

Because the relationship between HIF-1α and ADORA2B is just beginning to be understood, we assumed that dysregulation of ADORA2B is involved in progression of OSCCs via activation of HIF-1α. We also performed functional analysis to define the biologic effect of ADORA2B in OSCC.

## Methods

### Ethics statement

The Ethics Committee of the Graduate School of Medicine, Chiba University (approval number, 236) approved the study protocol, which was performed according to the tenets of the Declaration of Helsinki. All patients provided written informed consent.

### OSCC-derived cellular lines and tissue specimens

Immortalized human OSCC-derived cell lines (HSC-2, HSC-3, HSC-4, Ca9-22, Sa3, HO-1-u-1, HO-1-N-1, KOSC2, and SAS) were obtained from the Human Science Research Resources Bank (Osaka, Japan) or the RIKEN BioResource Center (Ibaraki, Japan) through the National BioResource Project of the Ministry of Education, Culture, Sports, Science and Technology (Tokyo, Japan). Short tandem repeat profiles confirmed cellular identity. All OSCC-derived cells were grown in Dulbecco’s modified Eagle medium/F-12 HAM (Sigma-Aldrich, St. Louis, MO, USA) supplemented with 10 % fetal bovine serum (Sigma-Aldrich) and 50 units/ml penicillin and streptomycin (Sigma-Aldrich). Isolated and primary-cultured human normal oral keratinocytes (HNOKs) were used as a normal tissue control [10-15]. They were isolated from healthy oral mucosal epithelial specimens from three young patients at Chiba University Hospital. The HNOKs were primary cultured and maintained in Keratinocyte-SFM (Invitrogen, Carlsbad, CA, USA) comprised of growth supplement (Invitrogen) and 50 units/ml penicillin and streptomycin (Sigma).

One hundred pairs of primary OSCC samples and corresponding normal oral epithelial tissues were obtained during surgeries at Chiba University Hospital. The resected tissues were fixed in 20 % buffered formaldehyde solution for pathologic diagnosis and immunohistochemistry (IHC). The Department of Pathology of Chiba University Hospital histopathologically diagnosed each OSCC sample according to the World Health Organization criteria [16]. Clinicopathologic staging was determined by the TNM (tumor-node-metastasis) classification of the International Union against Cancer [17]. All patients had OSCC that was confirmed histologically; the samples were checked to ensure that tumoral tissue was present in more than 80 % of the specimens.

### Preparation of cDNA and protein

Total RNA was isolated using Trizol Reagent (Invitrogen) according to the manufacturer’s instructions. cDNA was generated using 5 μg total RNA from OSCC-derived cell lines using Ready-To-Go You-Prime First-Strand Beads (GE Healthcare, Buckinghamshire, UK) and oligo (dT) primer (Hokkaido System Science, Sapporo, Japan) according to the manufacturer’s instructions. The cells were washed twice with cold phosphate-buffered saline (PBS) and centrifuged briefly. The cell pellets were incubated at 4 °C for 30 minutes in a lysis buffer (7 M urea, 2 M thiourea, 4 % w/v CHAPS, and 10 mM Tris pH 7.4) with a proteinase inhibitor cocktail (Roche Diagnostics, Mannheim, Germany). HNOKs proteins were extracted from the same samples obtained for RNA. The protein concentration was measured using the BCA Protein Assay Kit (Thermo, Rockford, IL, USA).

### mRNA expression analysis

Real-time quantitative reverse transcriptase-polymerase chain reaction (qRT-PCR) was performed using the LightCycler 480 apparatus (Roche Diagnostics). Primers and universal probes were designed using the Universal Probe Library (Roche Diagnostics), which specifies the most suitable set. The primer sequences used for qRT-PCR were: *ADORA2B*, forward, 5′-GGGCTTCTGCACTGACTTCT -3′; reverse, 5′-CCGTGACCAAACTTTTATACCTG -3′; and universal probe #34, and the glyceraldehyde-3-phosphate dehydrogenase (*GAPDH*) , forward, 5′-AGCCACATCGCTCAGACAC -3′; reverse, 5′-GCCCAATACGACCAAATCC -3′; and universal probe #60. The transcript amount for ADORA2B was estimated from the respective standard curves and normalized to the GAPDH transcript amount determined in corresponding samples. All samples were analyzed in triplicate, and three independent RNA preparations were analyzed from each cell line.

### Immunoblotting analysis

Protein extracts (20 μg) were separated by sodium dodecyl sulfate polyacrylamide gel electrophoresis in 4-12 % gel, transferred to nitrocellulose membranes, and blocked for 1 hour at room temperature in Blocking One (Nacalai Tesque, Tokyo, Japan). The membranes were incubated with rabbit anti-ADORA2B polyclonal antibody (Chemicon International, Temecula, CA, USA), mouse anti-α tubulin monoclonal antibody (Santa Cruz Biotechnology, Santa Cruz, CA, USA), mouse anti- HIF-1α monoclonal antibody (Santa Cruz Biotechnology), rabbit anti-Akt monoclonal antibody (Santa Cruz Biotechnology), rabbit anti-pAkt monoclonal antibody (Santa Cruz Biotechnology) , rabbit anti-ERK1/2 monoclonal antibody (Santa Cruz Biotechnology), and rabbit anti-pERK1/2 monoclonal antibody (Santa Cruz Biotechnology) overnight at 4 °C. The membranes were washed with 0.1 % Tween-20 in Tris-buffered saline, incubated with secondary antibody and coupled to horseradish peroxidase-conjugated anti-rabbit or anti-mouse IgG (Promega, Madison, WI, USA) for 1 hour at room temperature. The membranes were detected using SuperSignal West Pico Chemiluminescent substrate (Thermo), and immunoblotting was visualized by exposing the membranes to ATTO Light-Capture II (ATTO, Tokyo, Japan). Signal intensities were quantitated using the CS Analyzer version 3.0 software (ATTO).

### Transfection with shRNA plasmid

OSCC cellular lines (HSC-3 and -4) were transfected with ADORA2B shRNA (shADORA2B) or control shRNA (shMock) vectors (Santa Cruz Biotechnology) using Lipofectamine LTX and Plus Reagents (Invitrogen). After transfection, the stable transfectants were isolated by a culture medium containing 2 ng/mL Puromycin (Invitrogen). Two to three weeks after transfection, viable colonies were transferred to new dishes. shADORA2B and shMock cells were used for further experiments.

### Cellular growth

The transfectants were seeded in six-well plates at a density of 1 × 10^4^ viable cells/well. Cells were harvested for 168 hours and counted every 24 hours. We determined proliferation rates using the 3-(4, 5-dimethylthiazol-2-yl)-5-(3- carboxymethoxyphenyl)-2-(4-sulfophenyl)-2H-tetrazolium (MTS) assay (Promega).

### Apoptosis assay

Apoptosis detection was performed in transfected and control cells by staining with the Annexin V-FITC Apoptosis Detection kit (Abcam, Cambridge, MA, USA). Cells were harvested at a density of 2×10^5^ cells/ml in 1 × binding buffer and stained with FITC-labeled Annexin V and propidium iodide (PI) for 5 min at room temperature. Samples were immediately analyzed using the FACScaliber flow cytometer (Becton-Dickinson, San Jose, CA, USA).

### Hypoxic treatment

To create hypoxia, cells were placed for 12 to 48 hours in a modular incubator chamber (Astec, Fukuoka, Japan) and flushed with a gas mixture containing 1 % oxygen, 5 % carbon dioxide, and balance N2.

### IHC

IHC was performed on 4-μm sections of paraffin-embedded specimens using rabbit anti-ADORA2B polyclonal antibody (Chemicon International) and mouse anti- HIF-1α monoclonal antibody (Santa Cruz Biotechnology). Briefly, after deparaffinization and hydration, the endogenous peroxidase activity was quenched by a 30-minute incubation in a mixture of 0.3 % hydrogen peroxide solution in 100 % methanol; the sections were blocked for 2 hours at room temperature with 1.5 % blocking serum (Santa Cruz Biotechnology) in PBS before reacting with anti-ADORA2B antibody overnight at 4 °C in a moist chamber. Upon incubation with the primary antibody, the specimens were washed three times in PBS and treated with Envision reagent (DAKO, Carpinteria, CA, USA) followed by color development in 3,3′-diaminobenzidine tetrahydrochloride (DAKO). The slides then were counterstained slightly with hematoxylin, dehydrated with ethanol, cleaned with xylene, and mounted. Nonspecific binding of an antibody to proteins other than the antigen sometimes occurred. As a negative control, triplicate sections were immunostained without exposure to primary antibodies, which confirmed the staining specificity. To quantify the status of ADORA2B protein expression in those components, we used the IHC scoring systems described previously [13, 15, 18-20]. The mean percentages of positive tumoral cells were determined in at least three random fields at 400× magnification in each section. The intensities of the ADORA2B immunoreactions were scored as follows: 0+, none; 1+, weak; 2+, moderate; and 3+, intense. The cellular number and staining intensity were multiplied to produce an ADORA2B IHC score. Cases with an ADORA2B IHC score exceeding 120.0 (+3 standard deviation [SD] score for normal tissue) were defined as ADORA2B-positive. The ±3-SD cutoff, which statistically is just 0.2 % of the measurement and would be expected to fall outside this range, was used because it was unlikely to be affected by random experimental error produced by sample manipulation [21]. Two independent pathologists from Chiba University Hospital, neither of whom had knowledge of the patients’ clinical status, made these judgments.

### Statistical analysis

In comparisons of ADORA2B expression levels, statistical significance was evaluated using the Mann–Whitney U test. Relationships between ADORA2B-immunohistochemical staining scores and clinicopathological profiles were evaluated using the χ2 test, Fisher’s exact test, and Mann–Whitney U test. *p* < 0.05 was considered significant. The data are expressed as the mean ± standard error of the mean (SEM). Survival curves were obtained by the Kaplan–Meier method, and differences in survival rates between ADORA2B-positive and ADORA2B negative cases were compared by log-rank test with 95 % significance.

## Results

### Evaluation of ADORA2B expression in OSCC-derived cell lines

**Fig. 1:**
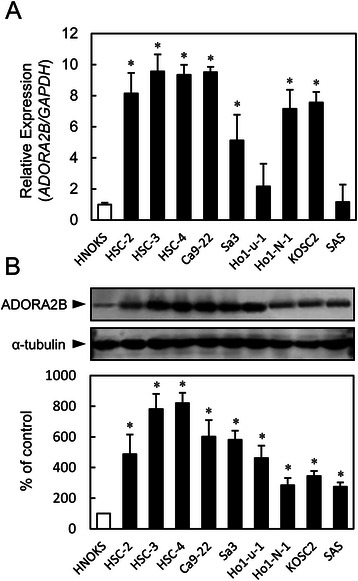
Evaluation of ADORA2B expression in OSCC-derived cell lines. **a** Quantification of *ADORA2B* mRNA expression in OSCC-derived cell lines by qRT-PCR analysis. Significant (*p* < 0.05, Mann–Whitney U test) up-regulation of *ADORA2B* mRNA is seen in seven OSCC-derived cell lines compared with the HNOKs. Data are expressed as the means ± SEM of triplicate results. **b** Representative immunoblotting analysis of ADORA2B protein in OSCC-derived cell lines and HNOKs. ADORA2B protein expression is up-regulated in OSCC-derived cell lines compared with the HNOKs. Densitometric ADORA2B protein data are normalized to α-tubulin protein levels. The values are expressed as a percentage of the HNOKs

To analyze the expression status of ADORA2B, we performed qRT-PCR and immunoblotting analysis using nine OSCC-derived cell lines (HSC-2, HSC-3, HSC-4, Cap-22, Sa3, Ho-1-u-1, Ho-1-N-1, KOSC2, and SAS) and HNOKs. *ADORA2B* mRNA was up-regulated significantly (*p* < 0.05) in seven OSCC-derived cell lines compared with the HNOKs (Fig. 1a). Figure 1b shows representative results of immunoblotting analysis. ADORA2B protein expression increased significantly in all OSCC-derived cell lines compared with the HNOKs. Expression analysis indicated that both transcription and translation products of this molecule were highly expressed in the OSCC-derived cell lines.

### Evaluation of ADORA2B expression in primary OSCCs

**Fig. 2:**
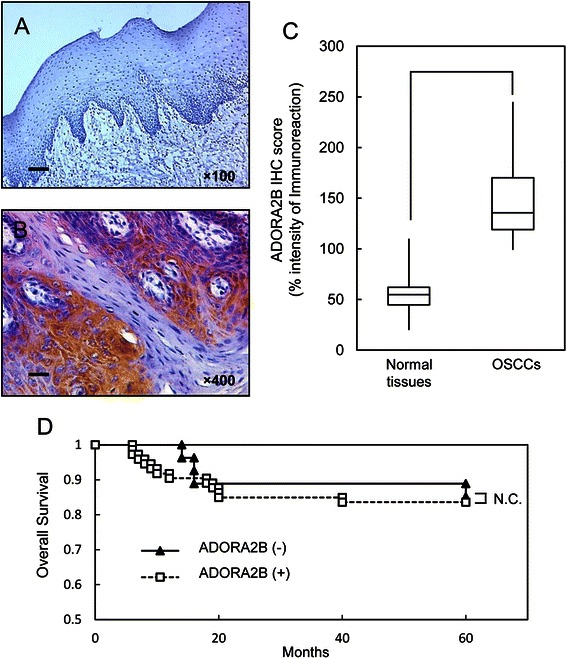
Evaluation of ADORA2B protein expression in primary OSCCs. **a**, **b** Representative IHC results of ADORA2B in normal oral tissue and primary OSCC. **a** Normal oral tissues show almost negative immunostaining. Original magnification, ×100. Scale bars, 50 mm. **b** ADORA2B-positive cases of OSCC. Positive immunoreaction for ADORA2B is detected in the cytoplasm. Original magnification, ×400. Scale bars, 10 mm. **c** The status of ADORA2B protein expression in primary OSCCs (*n* = 100) and the normal counterparts based on an IHC scoring system. IHC scores are calculated as follows: IHC score = 1 × (number of weakly stained cells in the field) + 2 × (number of moderately stained cells in the field) + 3 × (number of intensely stained cells in the field). The ADORA2B IHC scores for normal oral tissues and OSCCs range from 20 to 110 (median, 51) and 99 to 245 (median, 139.5), respectively. ADORA2B protein expression levels in OSCCs are significantly (*p* < 0.05, Mann–Whitney U test) higher than in normal oral tissues. **d** Kaplan–Meier survival curve for overall survival rates of patients with OSCCs based on the levels of ADORA2B expression. Up-regulated ADORA2B expression was not significantly related to overall survival rates (N.S. = *p* = 0.8037). The log-rank statistic was used to test the difference in survival times between the groups. ADORA2B (+), up-regulated ADORA2B; ADORA2B (−), down-regulated ADORA2B

To determine the expression status of ADORA2B in primary OSCCs and the relation to the clinicopathological characteristics, we investigated ADORA2B protein expression in primary OSCCs and paired normal oral tissues from 100 patients using the IHC scoring system. Representative IHC results for ADORA2B protein in the normal oral tissue and primary OSCC are shown in Fig. 2a-c. Positive staining was seen predominantly in the cytoplasm of primary OSCC samples (Fig. 2b). The ADORA2B IHC scores in primary OSCCs (range, 99-245; median, 139.5) were significantly higher than in normal tissues (20-110; median, 51) (Fig. 2c) (*p* < 0.05).

Table 1 shows the correlations between the clinicopathological characteristics of the patients with OSCC and the status of ADORA2B protein expression using the IHC scoring system. We defined cases with an IHC score exceeding 120 (+3-SD score for normal tissue) as ADORA2B-positive. Among the clinical parameters, ADORA2B expression was related significantly (*p* = 0.02) to the primary size of the OSCC tumors. Moreover, a significant (*p* = 0.01) difference was found in the ADORA2B expression levels between the T1/T2 group and the T3/T4 group. Survival analysis using the Kaplan–Meier method showed that ADORA2B expression was not significantly related to overall survival (*P* = 0.8037; Fig. 2d).

**Table 1:** Correlation between ADORA2B expression and clinical classification

Clinical classification	Total	Results of immunostaining		*p* value
		No. patients (%)		
		ADORA2B negative	ADORA2B positive	
Age at surgery (years)				
<60	27	7 (26)	20 (74)	0.95
≧60, <70	26	8 (31)	18 (69)	
≧70	47	13 (28)	34 (72)	
Gender				
Male	64	21 (33)	43 (67)	0.24
Female	36	7 (19)	29 (81)	
T-primary tumor				
T1	8	3 (38)	5 (62)	0.02*
T2	60	22 (37)	38 (63)	
T3	15	2 (13)	13 (87)	
T4	17	1 (6)	16 (94)	
T1 + T2	58	25 (43)	33 (57)	0.01*
T3 + T4	32	3 (10)	29 (90)	
N-regional lymph node				
N (negative)	58	14 (24)	44 (76)	0.39
N (positive)	42	14 (33)	28 (67)	
Stage				
I	7	3 (43)	4 (57)	0.54
II	38	11 (29)	27 (71)	
III	17	4 (24)	13 (76)	
IV	38	10 (26)	28 (74)	
Histopathologic type				
Well	64	15 (23)	49 (77)	0.25
Moderately	31	11 (35)	20 (65)	
Poorly	5	2 (40)	3 (60)	
Tumor site				
Gingiva	35	11 (31)	24 (69)	0.84
Tongue	50	12 (24)	38 (76)	
Buccal mucosa	9	2 (22)	7 (78)	
Oral floor	6	3 (50)	3 (50)	

*p* < 0.05

### Establishment of ADORA2B knockdown cells

**Fig. 3:**
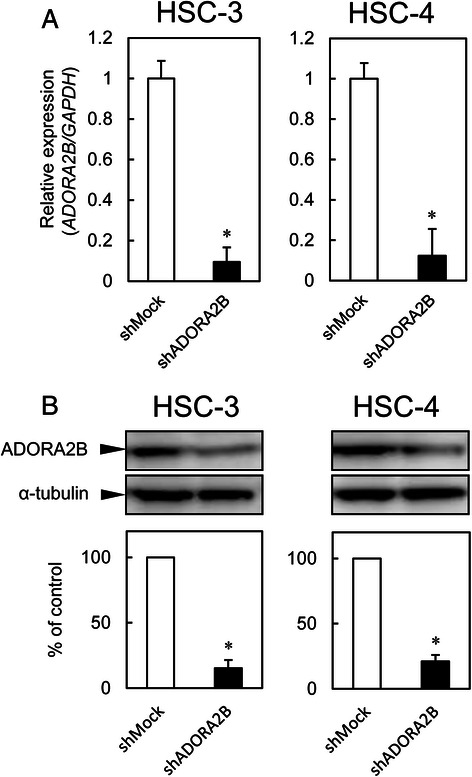
Expression of ADORA2B in shADORA2B-transfected cells. **a** Expression of *ADORA2B* mRNA in shMock- and shADORA2B-transfected cells (HSC-3- and -4-derived transfectants). The *ADORA2B* mRNA expression in the shADORA2B is significantly (*p* < 0.05, Mann–Whitney U test) lower than that in the shMock-transfected cells. **b** Immunoblotting analysis of ADORA2B protein in the shMock- and shADORA2B-transfected cells (HSC-3- and -4-derived transfectants). The ADORA2B protein in the shADORA2B transfected cells is decreased markedly compared with the shMock-transfected cells

The OSCC-derived cell lines (HSC-3 and -4) were transfected with ADORA2B shRNA (shADORA2B) and the control shRNA (shMock). qRT-PCR and immunoblotting analyses were performed to assess the efficiency of ADORA2B knockdown. *ADORA2B* mRNA expression in shADORA2B-transfected cells was significantly (*p* < 0.05) lower than in shMock-transfected cells (Fig. 3).

### Reduced cellular growth in ADORA2B knockdown cells

**Fig. 4:**
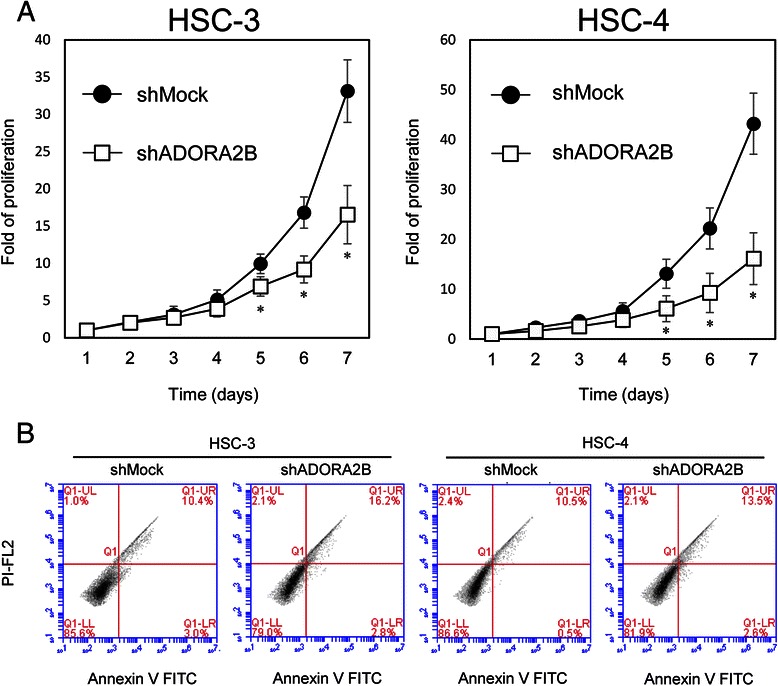
Proliferation and apoptosis assay of shADORA2B-transfected cells. **a** To determine the effect of shADORA2B on cellular proliferation, shADORA2B- and shMock-transfected cells were seeded in six-well plates at a density of 1 × 10^4^ viable cells/well. The shADORA2B transfected HSC-3 and -4 cells have significantly (*p* < 0.05, Mann–Whitney U test) decreased cellular growth compared with the shMock-transfected cells. **b** The apoptosis rate was assayed using flow cytometry after staining with Annexin V-FITC and propidium iodide

To study the effect of ADORA2B knockdown on cellular growth, we performed a cellular proliferation assay. shADORA2B and shMock cells were seeded in six-well plates at a density of 1 × 10^4^ viable cells/well that were counted for 168 hours. Both HSC-3 and -4 shADORA2B cells had a significant (*p* < 0.05) decrease in cellular growth compared with the shMock cells (Fig. 4a). To confirm apoptotic activity, shADORA2B and shMock cells were analyzed for Annexin V expression by flow cytometry. There was no significant difference in the percentage of annexin-V and PI positive cells in the shADORA2B and shMock cells (Fig. 4b).

### Hypoxic induction of ADORA2B

**Fig. 5:**
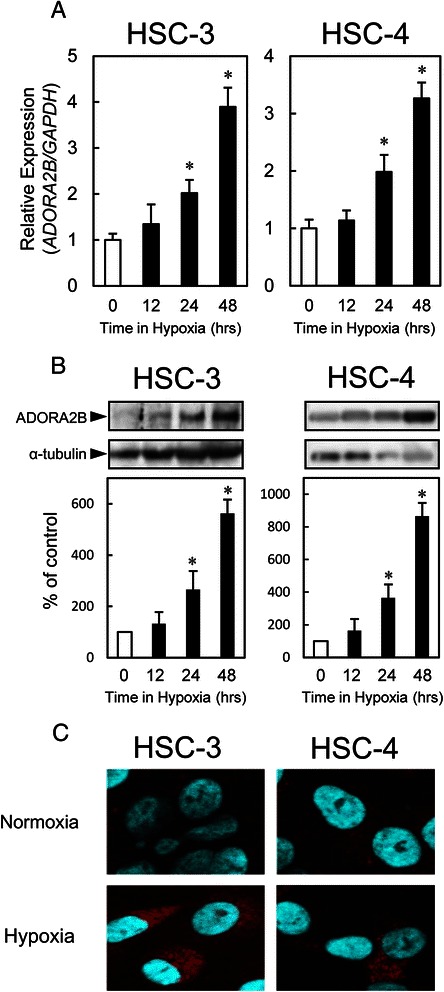
Hypoxic induction of ADORA2B. **a** OSCC-derived cell lines are exposed to hypoxia (1 % oxygen ) and probed for ADORA2B expression by qRT-PCR. Expression of *ADORA2B* mRNA is induced significantly (*p* < 0.05, Mann–Whitney U test) by hypoxia in OSCC-derived cell lines (HSC-3 and -4). **b** Immunoblotting analysis shows that the ADORA2B protein levels in hypoxia also have increased markedly compared with that in normoxia. **c** Immunocytofluorescence labeling of ADORA2B (*red*). Cellular nuclei are labeled with DAPI (*blue*). Immunocytofluorescence shows that the ADORA2B expression levels are higher in hypoxia than in normoxia in OSCC-derived cell lines (HSC-3 and -4)

To investigate the relationship between ADORA2B expression and oxygen supply, we examined ADORA2B expression under both normoxic and hypoxic conditions in the OSCC-derived cell lines (HSC-3 and -4, with higher ADORA2B protein expression). The expression of ADORA2B mRNA and protein were up-regulated significantly (*p* < 0.05) in these cell lines after 24 hours of hypoxia compared with a normoxia (Fig. 5a, b). Immunocytofluorescence showed that the ADORA2B expression levels were higher in hypoxia than in normoxia in these cell lines (Fig. 5c).

### Activation of HIF-1α

**Fig. 6:**
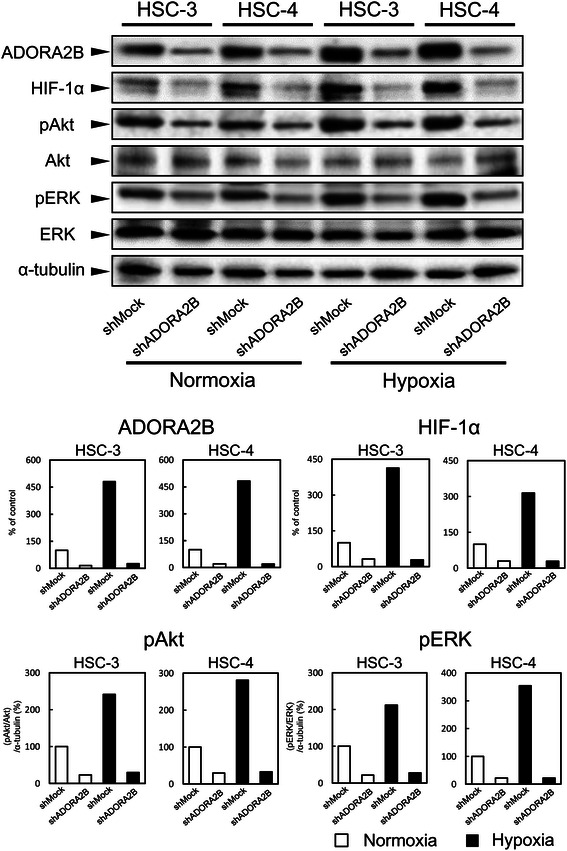
ADORA2B knockdown inhibits HIF-1α, Akt, and ERK1/2 activation. Immunoblotting analysis of ADORA2B, HIF-1α, pERK1/2, and pAkt proteins in the Mock and shADORA2B cells under both normoxic and hypoxic conditions. The expression of ADORA2B, HIF-1α, pERK1/2, and pAkt protein were up-regulate in the shMock cells of hypoxia compared with a normoxia. The HIF-1α protein levels in the shADORA2B-transfected cells (HSC-3- and -4-derived transfectants) also have decreased markedly compared with that in the shMock-transfected cells under both normoxic and hypoxic conditions. ADORA2B knockdown causes decreased levels of pAkt and pERK1/2 compared with the shMock-transfected cells (HSC-3- and -4-derived transfectants); the AKT and ERK1/2 protein levels are unchanged under both normoxic and hypoxic conditions. Densitometric ADORA2B, HIF-1α, pAkt/Akt and pERK/ERK protein data were normalized to the α-tubulin protein levels

To investigate a potential underlying mechanism of reduced cellular proliferation in the shADORA2B-transfected cells, we assessed the HIF-1α pathways. ADORA2B knockdown causes markedly decreased protein expression levels of HIF-1α compared with shMock-transfected cells (Fig. 6). To evaluate the status of the protein expressions of ERK1/2 phosphorylation (pERK1/2) and Akt phosphorylation (pAkt), we performed immunoblotting analyses in the shADORA2B transfected cells. ADORA2B knockdown caused markedly decreased levels of pERK1/2 and pAkt compared with shMock-transfected cells. Results from immunoblotting analyses indicated that the phosphorylation levels of ERK1/2 and Akt decreased, whereas ERK1/2 and Akt proteins were unchanged (Fig. 6). Moreover, to evaluate the status of the protein expressions of HIF-1α, pERK1/2, and pAkt under hypoxic conditions, we performed immunoblotting analyses in the shMock cells and shADORA2B-transfected cells. HIF-1α, pERK1/2, and pAkt expression levels were higher in hypoxia than in normoxia. During a hypoxic conditions, the levels of HIF-1α, pERK1/2, and pAkt protein were not increased significantly in the shADORA2B-transfected cells (Fig. 6). In addition to the in vitro data, we also investigated the correlation of ADORA2B and HIF-1α in ADORA2B-positive clinical samples by IHC staining. The high expression of HIF-1α protein was detected in 19 out of 20 these samples (Additional file 1).

## Discussion

There is growing evidence that adenosine receptor agonists are attractive therapeutic targets for a number of conditions, including inflammation and certain cancer types [1, 22-27]. Adenosine receptors are found to be up-regulated in various tumoral cells [28]. ADORA2B has important roles in cellular proliferation and differentiation and apoptosis of tumoral cells [29-31]. Our previous microarray data showed significant up-regulation of ADORA2B in OSCC-derived cell lines [32]. The current study found a high prevalence of increased ADORA2B expression in patients with OSCC and a significant correlation with the primary tumoral size (Table 1). These findings indicated that ADORA2B overexpression may be linked to human oral carcinogenesis, has an important role in OSCC development, and is related closely to tumoral progression. However, the detailed function of ADORA2B in OSCC remains unclear. To determine whether ADORA2B function is relevant to OSCC progression, we performed functional analysis to define the biologic effect of ADORA2B.

A significant up-regulation of ADORA2B was detected in all OSCC-derived cells examined at protein levels (Fig. 1b), indicating that ADORA2B expression is necessary for oral carcinogenesis. On the other hand, the mRNA expression states depended on the cells (Fig. 1a). A possible explanation for this discrepancy is that ADORA2B proteins are differently affected by the post-translational ubiquitination and proteolysis in each tumor cell lines. In this context, discrepancies between mRNA expression levels and protein levels has been reported in OSCC cellular lines [33]. Many studies have indicated that ADORA2B plays critical roles, including tumoral development and progression, in cancers [3, 5-7, 31, 32]. In contrast, some studies have reported that ADORA2B has no role in adenosine-induced proliferation of human melanoma cells [34]. ADORA2B was up-regulated consistently in colorectal carcinoma tissues and colon cancer cell lines compared with normal colorectal mucosa [6].

The current study showed that hypoxia was involved with ADORA2B up-regulation in OSCC cells (Fig. 5a, b). Most solid tumors do not receive sufficient oxygen and tumoral cells grow in hypoxia [6]. Hypoxia promotes release of vascular endothelial growth factor by increasing ADORA2B expression and decreasing ADORA2A expression in human endothelial and smooth muscle cells [35].

Intratumoral hypoxia and genetic alterations lead to HIF-1α overexpression, which has been associated with increased patient mortality in several cancer types [36]. To date, expression of ADORA2B correlation with HIF-1α expression have not been elucidated in OSCC tissues. Our data showed that the immunoexpression of ADORA2B was correlated with HIF-1α expression in primary OSCCs (Additional file 1). HIF-1α is a common transcription factor induced by ADORA2B activation in inflammatory bowel disease and urologic diseases [37, 38]. Based on these findings, we observed the expression status of HIF-1α in the ADORA2B knockdown cells. As expected, HIF-1α was down-regulated significantly in the ADORA2B knockdown cells. We assessed the ERK1/2 and Akt pathways, which are up-regulated frequently in other cancers [7, 39], through HIF-1α activity and found that the phosphorylation levels of ERK1/2 and Akt decreased, whereas ERK1/2 and Akt proteins were unchanged. Moreover, results from immunoblotting analyses indicated that hypoxia promotes the up-regulation of HIF-1α, pERK1/2, and pAkt by increasing ADORA2B expression (Fig. 6). ERK1/2 and Akt activities are required by ADORA2B receptor activation [7]. The ADORA2B antagonist MRE 2029 F20 reduced ERK1/2 and Akt phosphorylation levels in melanoma cells in hypoxia [39].

## Conclusion

The current results indicated that ADORA2B is overexpressed frequently in OSCCs and may be associated strongly with tumoral progression. During hypoxia, there was significant up-regulation of ADORA2B expression in OSCC-derived cell lines. While further studies are needed to determine the interaction between ADORA2B and HIF-1α, the current data indicated that ADORA2B may be an efficacious treatment target in progression of primary OSCCs.

## Additional file

Additional file 1: **Evaluation of ADORA2B and HIF-1α expression in primary OSCCs.** (A-D) Representative IHC results of ADORA2B and HIF-1α in the same OSCC samples. (A, B) ADORA2B-positive cases of OSCC. Positive immunoreaction for ADORA2B is detected in the cytoplasm. Original magnification, ×100 (A) and × 400 (C). (C, D) HIF-1α-positive cases of OSCC. Positive immunoreaction for HIF-1α is detected in the nuclear and cytoplasm. Original magnification, ×100 (C) and × 400 (D). (TIFF 4229 kb)

## Abbreviations

ADORA2B: Adenosine A2b receptor
ERK1/2: Extracellular regulated kinase 1/2
HIF-1α: Hypoxia-inducible factor1α
HNOKs: Human normal oral keratinocytes
IHC: Immunohistochemistry
OSCC: Oral squamous cell carcinoma
qRT-PCR: Real-time quantitative reverse transcriptase-polymerase chain reaction
