# Supplementary figures and images for: Adenosine A2b receptor promotes progression of human oral cancer
Source: BMC Cancer. 2015 Jul 31;15:563. doi: 10.1186/s12885-015-1577-2 (PMC4520274; doi:10.1186/s12885-015-1577-2)

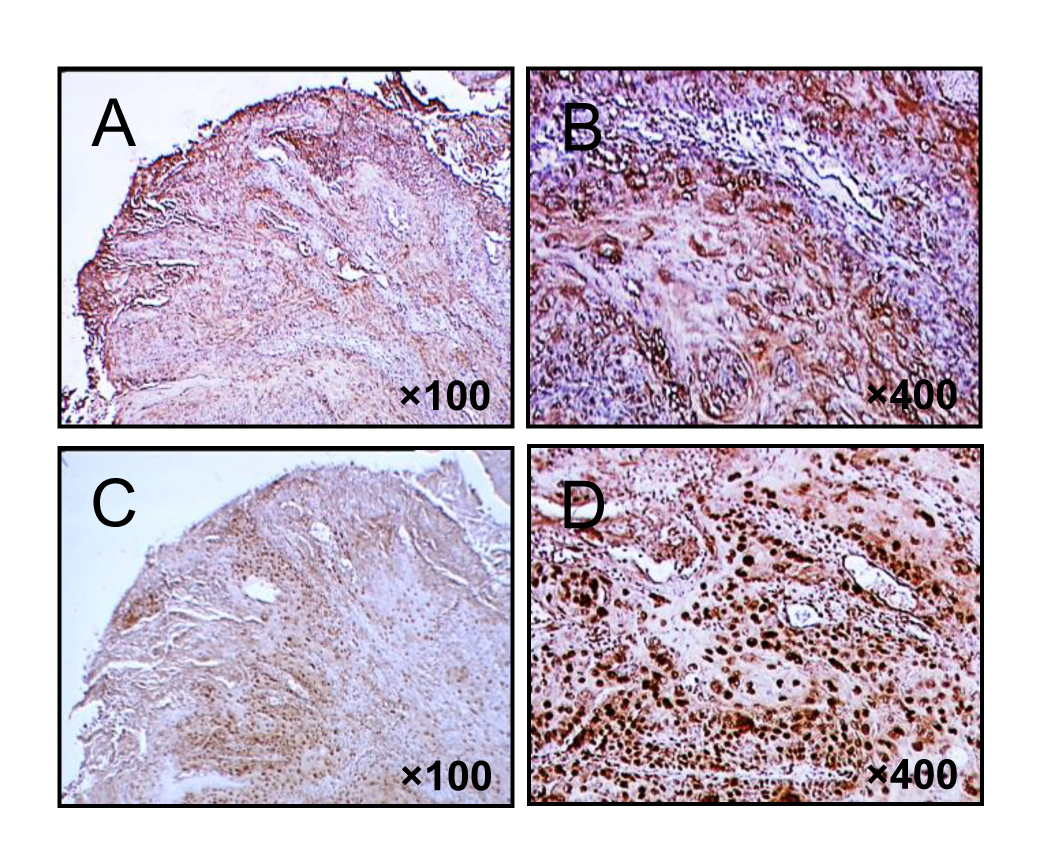

Supplement: Additional file 1: — Evaluation of ADORA2B and HIF-1α expression in primary OSCCs. (A-D) Representative IHC results of ADORA2B and HIF-1α in the same OSCC samples. (A, B) ADORA2B-positive cases of OSCC. Positive immunoreaction for ADORA2B is detected in the cytoplasm. Original magnification, ×100 (A) and × 400 (C). (C, D) HIF-1α-positive cases of OSCC. Positive immunoreaction for HIF-1α is detected in the nuclear and cytoplasm. Original magnification, ×100 (C) and × 400 (D). (TIFF 4229 kb) [file 12885_2015_1577_MOESM1_ESM.tiff]
